# Definitive Screening Design and Artificial Neural Network for Modeling a Rapid Biodegradation of Date Palm Fronds by a New *Trichoderma* sp. PWN6 into Citric Acid

**DOI:** 10.3390/molecules26165048

**Published:** 2021-08-20

**Authors:** Maha S. Elsayed, Noha M. Eldadamony, Salma S. T. Alrdahe, WesamEldin I. A. Saber

**Affiliations:** 1 Central Laboratory of Date Palm Research and Development, Agricultural Research Center, Giza 12112, Egypt; mahasobhy1000@yahoo.com; 2 Seed Pathology Department, Plant Pathology Institute, Agricultural Research Center, Giza 12112, Egypt; nohamohamadt@gmail.com; 3 Department of Biology, Faculty of Science, University of Tabuk, Tabuk 47731, Saudi Arabia; salrdahe@ut.edu.sa; 4 Microbial Activity Unit, Microbiology Department, Soils, Water and Environment Research Institute, Agricultural Research Center, Giza 12112, Egypt

**Keywords:** machine learning, organic acids, phosphate-solubilizing fungi, bioremediation, cellulase, glucose, date palm leaflet

## Abstract

Generally, the bioconversion of lignocellulolytics into a new biomolecule is carried out through two or more steps. The current study used one-step bioprocessing of date palm fronds (DPF) into citric acid as a natural product, using a pioneer strain of *Trichoderma* *harzianum* (PWN6) that has been selected from six tested isolates based on the highest organic acid (OA) productivity (195.41 µmol/g), with the lowest amount of the released glucose. *Trichoderma* sp. PWN6 was morphologically and molecularly identified, and the GenBank accession number was MW78912.1. Both definitive screening design (DSD) and artificial neural network (ANN) were applied, for the first time, for modeling the bioconversion process of DPF. Although both models are capable of making accurate predictions, the ANN model outperforms the DSD model in terms of OA production, as ANN is characterized by a higher value of R^2^ (0.963) and validation R^2^ (0.967), and lower values of the RMSE (13.44), MDA (11.06), and SSE (9749.5). Citric acid was the only identified OA as was confirmed by GC-MS and UPLC, with a total of 1.5%. In conclusion, DPF together with *T. harzianum* PWN6 is considered an excellent new combination for citric acid biosynthesis, after modeling with artificial intelligence procedure.

## 1. Introduction

The full utilization of natural resources is of great interest in maintaining sustainable social development. That is why there has been an increasing trend toward more efficient utilization of plant residues coinciding with releasing vast amounts of such value-added biomass into the ecosystem each year [1]. The most promising approach is the bioremediation of plant residuals into useful biomolecules.

After coconut and oil palms, date palm trees (*Phoenix dactylifera*), family *Palmae* (*Arecaceae*), is one of the most widespread palm species in the global agricultural industry [2]. There are nearly more than 100 million date palm trees worldwide [3], grown in tropical and subtropical regions, concentrated mainly in Egypt, Saudi Arabia, Iran, the UAE, and Algeria [4]. An average of 12–15 new date palm fronds (DPF) is formed, and therefore, the exact amount is removed as part of the maintenance of the palm [5], generating tons of DPF [6]. Although they can be converted to compost and/or used for traditional art, in most cases, they are burned, causing pollution to the environment. Alternatively, this large amount of biomass residues can be used in various biotechnological applications, such as the biosynthesis of biomolecules, e.g., organic acids, which was the target topic of the current investigation.

Nature has been enriched with microorganisms that can degrade complex biomass to simpler molecules through enzymatic action. The majority of bioconversion processes pass through intermediate step(s), consuming more time and effort. Direct conversion of plant biomass is not an exception. Therefore, finding out a microorganism that can directly convert, for example, DPF biomass into OA, without an intermediate of pentoses or hexoses, is of great importance. This is the first target of the current work.

Owing to their functional group, organic acids are exceptionally useful in diverse fields such as agricultural, pharmaceutical, food preservatives, pH adjusters, sweetness enhancers, leavening agents, and stabilizers [7,8,9]. Organic acids are important weak acid molecules that can be classified into two groups: the first has a long biosynthetic pathway, and the second has a short pathway; both groups are delivered from glucose [10]. Glucose is the vital base for the bioconversion process. The most significant commercial amount of fungal citric acid, for instance, is prepared by the fermentation of glucose or sucrose [8,11,12]. Alternatively, DPF could be biotechnologically attractive, because of its high content of glucose, which, unfortunately, occurs in a complex form. Therefore, it is better to be directly converted in single-step fermentation by a suitable microorganism into OA. To the best of our knowledge, there has been no previous report on the direct conversion of DPF, as a substrate, into OA production.

Solid-state or solid-substrate fermentation (SSF) is a biomolecule synthesis technique that involves inoculating a solid substrate with a suitable microorganism(s) in the absence or near absence of free-flowing water [13]. Compared to the submerged fermentation technique, the technology of SSF has several merits; of these, no obligation for complex machinery is required that lowers the energy demand, increases volumetric productivity, and raises the concentration and assembly permanence of the target molecule. Additionally, SSF can be used to ferment the cheap residuals that result from the industrial and/or agricultural activities in a relatively compacted fermentation space with a lower water activity, which also minimizes the contamination opportunity [13,14,15]. Therefore, SSF gained attention in the manufacturing of numerous microbial biomolecules.

Statistical screening designs are appealing in identifying the relative influence of a large number of factors, in a reasonably small-sized experiment, on a response of interest. Most screening designs limit each factor to only two levels, thus focusing on estimating the main effects only and not allowing the assessment of the curvature between the factor and the response. Therefore, the second step of experiments is required for capturing the curvature by adding middle levels [16,17]. The definitive screening design (DSD) is a new, improved class of three-level designs that was proposed recently to provide efficient estimates of main effects that are unbiased to any quadratic effects and two-factor interactions [16,18]. Unlike traditional screening by two-level fractional factorial designs, DSD may render follow-up experiments unnecessary in many situations, and further, it avoids the confounding of effects, and it can identify factors having a nonlinear or curvilinear effect on the response [16,17,18].

Artificial neural network (ANN) is the main component of artificial intelligence and one of the main tools used in machine learning, which is designed for stimulating the human brain in analyzing and processing information. This computational model is built with interconnected nodes within the hidden layer(s) capable of learning patterns and making decisions based on a piece of experimental data without any need to add further information [19,20]. 

The steps included in the ANN modeling procedure are the choice of network architecture, establishing of the hidden layer(s) with enough neurons, learning, training, and, finally, validation and verification of the ANN model. The learning mechanism is called backpropagation; i.e., the ANN tries to diagnose the diverse patterns in the data and compares the actual output with the desired one to detect any differences, which are dimensioned, through backpropagation, to achieve the desired output. During such intelligent backpropagation, the ANN is trained to generate precisely the desired output model that achieves the target. Therefore, this deep learning process is hypothetically more precise and can efficiently replace the other modeling approaches [19,20,21].

Owing to the lack of knowledge and previous studies on the modeling of the biodegradation process, the current work is a trial for sharing a new perspective approach in this field. Especially when knowing that the modern DSD is rarely used in the biological sciences, in general, and, to the best of our knowledge, this is the first study in which DSD was utilized to optimize a biomolecule manufacturing process. Furthermore, despite the recent various successful biotechnological applications of ANNs, there is no knowledge on modeling the biomolecules production from DPF using ANN. The current investigation was undertaken to share some knowledge about such processes.

## 2. Results

The start point in the current study was to select concisely and precisely the suitable microorganism that can efficiently convert plant residual biomass i.e., DPF into OA in a single step.

### 2.1. The Biodegradability of DPF by Trichoderma spp. 

Assuming equal biodegradation capabilities of DPF (null hypothesis, H_0_) by the tested *Trichoderma* spp., a comparison study was initiated to explore their ability to ferment DPF-based medium into organic acids. For such a target, the profile of cellulose-degrading capacity of the investigated fungi was, firstly, differentiated (Table 1). That preliminary step was required to test the possibility of a fungus to initiate and establish a good growth on the complex residual.

Although each *Trichoderma* sp. has a unique enzymatic pattern, there are some similarities between all fungal isolates, which were the ability to secrete substantial amounts of various cellulases that can degrade both cellulose (by filter-paperase (FPase), carboxymethyl cellulase (CMCase), and β-glucosidase) and hemicellulose as well (by xylanase). However, *Trichoderma* sp. PWN2 and PWN4 are exceptions regarding the negative CMCase production. Another similar point was the notable glucose monomers released in the hydrolysate of the fermented DPF. On the other side, there was a significant variation among fungal isolates regarding OA production, which varied from none to 195.41 µmol/g DPF; the same variation trend could be drawn to soluble phosphorus (P). Therefore, the H_0_ is false and rejected, consequently, it is replaced with the accepted alternative hypothesis (H_1_). 

All these results lead to studying the nature of the relationships among all tested parameters employing a simple correlation coefficient (*r*). The correlation between every pair of the tested criteria was compared at probability (*p*) ≤ 0.05. The amount of released glucose and xylanase did not show a significant correlation with the other tested parameters. The most apparent relationship was between OA production and filtrate pH, showing significant negative *r*, being −0.834 at *P* ≤ 0.05, and between OA production and soluble P, which were positively and significantly correlated (*r* = 0.935 at *p* ≤ 0.05). Despite having a sufficient active enzymatic profile, *Trichoderma* sp. PWN6 showed the lowest amount of the released glucose. However, this isolate was selected as the potent OA producer to model the bioconversion process of DPF into OA.

### 2.2. Screening and Optimizing the Fermentation Criteria Using DSD

Seven independent factors i.e., steam-exploded biomass (SEB), DPF size, incubation time, incubation temperature, inoculation size, tricalcium phosphate (TCP), and (NH_4_)_2_SO_4_ (AS), were screened for their possible effect on OA biosynthesis by the selected *Trichoderma* sp. PWN6. The experiments were performed following the matrix of DSD (Table 2), assuming an equal effect of the seven tested independent variables, implying, also, that there is no association between the variables and the OA production (H_0_). Various combinations were investigated, and the experimental results obtained from DSD were statistically analyzed to explore which factor(s) exerts a feasible effect on OA formation by the selected fungus. The results reveal apparent variation in OA response among the various runs, yet the predicted values of the OA are relatively close to the experimental values.

**Figure 1:**
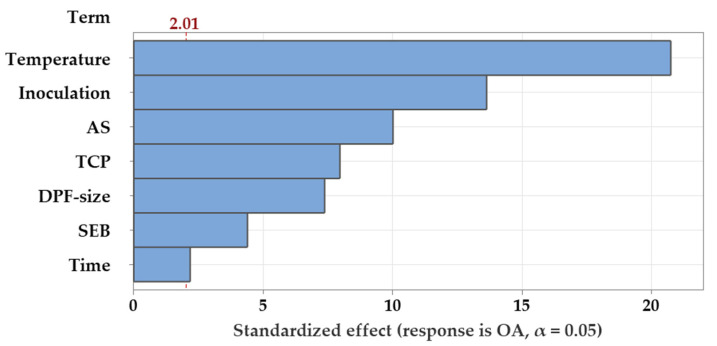
Pareto chart, displaying the standardized effects of each of the tested independent variables on the OA production by *Trichoderma* sp. PWN6. AS, (NH_4_)_2_SO_4_; TCP, tricalcium phosphate, SEB, steam-exploded biomass of DPF.

A Pareto chart of the standardized effects was generated (Figure 1) to determine which independent variables contribute to the variability in the OA production. The relative magnitude and the absolute value of the standardized effects of the tested factors are figured in descending order. All the tested factors extended the farthest and surpassed the threshold of the reference line (2.01), which is the minimum threshold value of the standardized effect at the corresponding significance level (*p* ≤ 0.05), recording a significant effect on OA production.

For further assessment of the null hypothesis, data (Table 3) display the significant influence of the tested independent variables at *p* ≤ 0.05. The values of the regression coefficients for each of the tested salts were calculated and found to vary from positive to negative values. The ANOVA showed that the model term is statistically significant (*p* < 0.001); therefore, the association between the OA and each factor in the design was compared with the corresponding *p*-value of each term. Variable(s) associated with OA at *p* ≤ 0.05 is considered statistically significant. Again, all tested parameters have a statistically significant association with OA, contributing to 95.13% of the model variability. However, as indicated by the regression coefficient, steam-exploded biomass (SEB) and incubation temperature had a positive impact, whereas the others had a negative one.

The fitting statistics measured the aptness of the model data, in which the standard deviation was estimated to be 16.7051. The values of coefficient of determination (R^2^) show reasonably high values, being 0.951, 0.944, and 0.933 for R^2^, adjusted-R^2^, and predicted-R^2^, respectively. Nevertheless, the H0 was rejected, and H1 was accepted since all parameters showed a significant effect; hence, they were subjected to further evaluation.

**Figure 2:**
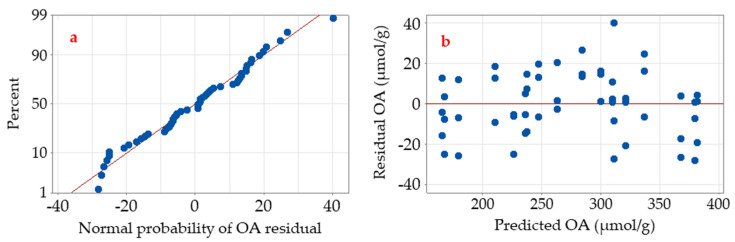
The normal probability of residuals (**a**) and the predicted vis residual (**b**) plots of the experimental DSD data of OA production by *Trichoderma* sp. PWN6.

The adequacy of the assumptions of the analysis of DSD data was checked utilizing the residual analysis. Plotting the normal probability plot of the residuals (Figure 2a) shows that the residuals of OA production are normally distributed and follow approximately a straight line. The frequency of the residuals was plotted. The predicted vis residual histogram (Figure 2b) indicates that the residuals (and hence the error terms) are distributed randomly but evenly along both 0-axis sides, with just one extreme outlier point (greater than 40). Accordingly, the regression equation in coded units was generated to be:

Total OA (µmol/g) = 273.32 + 10.49 (SEB) − 19.17 (DPF size) − 5.65 (Time) + 53.93 (Temperature, °C) − 35.44 (Inoculation size) − 20.70 (TCP) − 26.03 (AS)

Based on the experimental design of DSD and data analysis, the conclusion that can be drawn is that the operation conditions, including the seven tested independent variables, were significant and effective on OA production. So, the design matrix of DSD and their data were further modeled using the ANN. 

### 2.3. Modeling OA Biosynthesis Using ANN

The predictive ANN model was constructed using a fully connected neural network platform with a multilayer feed-forward ANN architecture to model the OA production by *Trichoderma* sp. PWN6. Numerous hidden neurons, ranging from 3 to 10, and various combinations of ANN-specific parameters, such as learning rate (0.1), were examined to identify the optimum architectural structure and the optimal number of neurons in the hidden layer. All nodes share the hyperbolic tangent sigmoid activation function in the hidden layer (NTanH).

**Figure 3:**
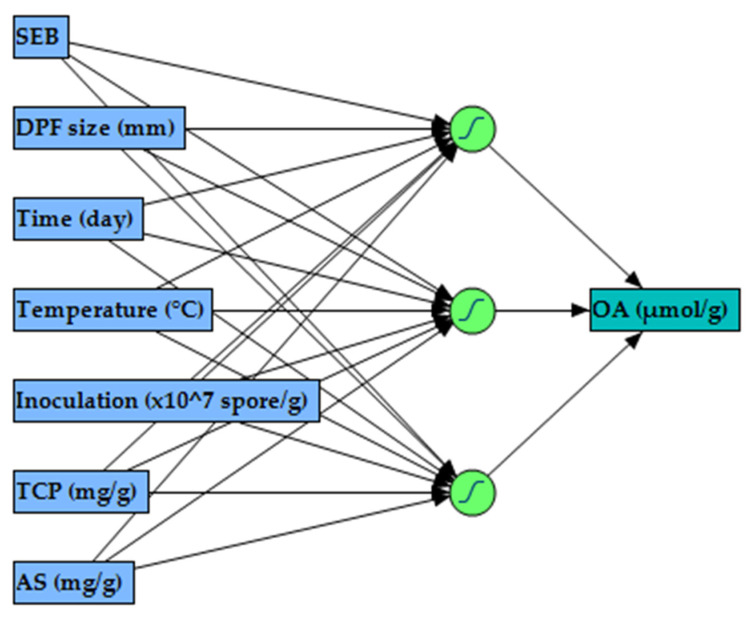
The artificial neural network’s general layout consists of one input layer with seven neurons, a hidden layer with three neurons, and an output layer with one neuron.

The ANN training was performed, employing the holdback procedure at a proportion of 0.3333, which randomly portioned DSD data into training, using 36 runs and validation using 18 runs. After several learning trials, each of 100 tours, one hidden layer with three neurons, NTanH(3), with a learning rate of 0.1, using the squared penalty method was found to have the maximum performance in modeling OA production. The ANN topology (Figure 3) was constructed in three layers, which was designated as 7-3-1. The input layer comprised of seven neurons (SEB, DPF size (mm), incubation time (day), incubation temperature (°C), inoculation (spore/g), TCP (mg/g), and AS (mg/g)) that is determined by the number of the examined independent factors. The output layer, with the hyperbolic tangent sigmoid activation function, has one neuron (OA, µmol/g), representing the response factor. The in-between single hidden layer performed better when using three hidden neurons. 

The ANN model’s generality was tested by reducing errors during training and validation. The network was trained until the R^2^ was maximized. The machine learning and validation processes were performed on the constructed ANN with a trial-and-error procedure. The trained network’s performance was evaluated based on the neural network’s ability to anticipate outputs comparable to or extremely near the response target value, with values of R^2^ = 96.04 and 96.71 for the 36 training runs and the 18 validation runs, respectively. Once the validation subset was selected, 100 tours of training were performed. The ANN predicted values of each resulting point of the DSD data were computed and shown with the anticipated DSD and experimental values (Table 2). ANN predicted values, and their errors showed reasonable agreement with the experimental ones and showed lower residual values than those obtained by the DSD model.

**Figure 4:**
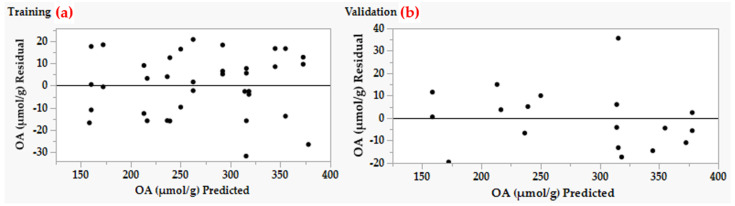
Residuals versus ANN predicted values OA production by *Trichoderma* sp. PWN6.

To assess and explore the adequacy and fitness of the ANN training and validation process to predict OA production, the values of R^2^ for the training and validation processes were found to be 0.960 and 0.967, respectively. Furthermore, the values of residual versus predicted values by the model were plotted. The plot (Figure 4a,b) depicts an even spread of the residual data above and below the 0-axis. These patterns are ideal enough to support the adequacy of the ANN model.

### 2.4. Fitness Comparison of DSD and ANN Models

The overall model performance by both DSD and ANN was tested and compared based on the model’s ability to correctly classify the fitness of OA production by the two generated models. The statistical parameters used to assess and evaluate the accuracy of both models were calculated (Table 4). R^2^, root mean squared error (RMSE), and the mean absolute deviation (MAD) statistics were calculated for training, validation, and testing sets of DSD and ANN. Higher values of R^2^ were observed for the training, validation, and testing of the ANN model compared to the DSD model. In contrast, RMSE and MAD recorded lower values. Comparing the overall performance of both models shows the same previous trend for all the tested statistics, including higher R^2^ value and lower RMSE, MAD, and the sum of squares due to error (SSE) of ANN compared to DSD, concluding that the ANN model is slightly better at the overall classification statistics.

**Figure 5:**
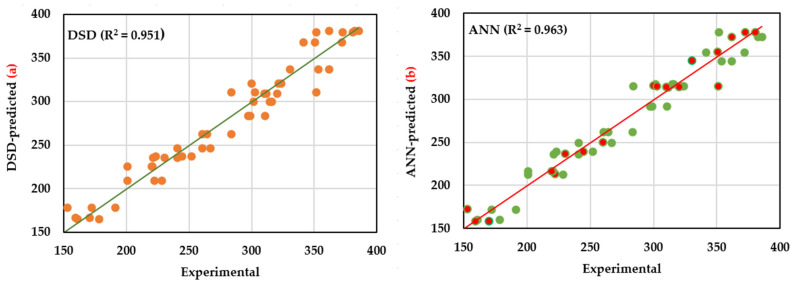
Actual vis DSD and ANN predicted values for OA production by *Trichoderma* sp. PWN6. The red points on the ANN graph are the eighteen random points that were used for the validation of the ANN model, while the 36 green points were utilized for training.

Likewise, the predicted values by both models were plotted against the corresponding actual (experimental) values to compare the fitness of both models (Figure 5a,b). Again, the ANN model predicts substantially closer points to the line of perfect prediction than the DSD model in the linear regression study. As a result, the ANN model outperforms the DSD model in terms of generalization capability.

### 2.5. Experimental Validation of Both Models

**Figure 6:**
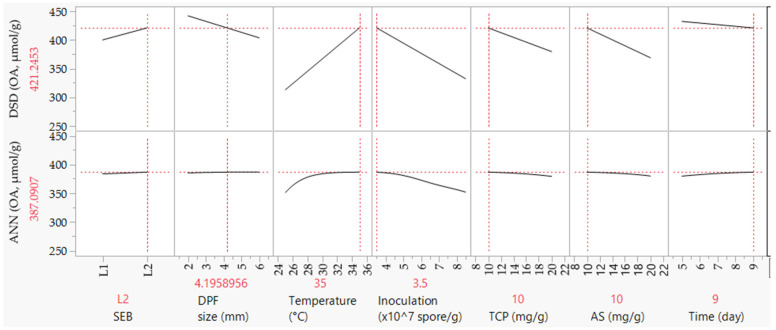
The theoretical values of the tested parameters and the corresponding predicted OA yields, which were estimated based on DSD and ANN models.

The response maximization of OA production was carried out to determine the optimal combination of the tested variables. DSD and the well-learned ANN models were evaluated regarding their capacity to forecast the production of OA by *Trichoderma* sp. PWN6 under laboratory conditions. First, the prediction model was used to calculate the theoretical values of the seven tested variables. Figure 6 shows the pattern of every single factor while keeping the other six factors constant; as shown, the theoretical level of the seven variables was found to yield theoretical values of 421.25 and 387.09 µmol OA/g based on the prediction model of DSD and ANN, respectively. The variation in OA values among actual and both DSD and ANN is simply due to the different models used for the calculation of the predicted OA. These levels of variables and their response were validated under the laboratory to check the applicability of the model. The experimental value was 391.37 ± 1.38 µmol OA/g; this value is more obeyed and closer to that estimated by the ANN model.

### 2.6. Specification of Components Using Gas Chromatography-Mass Spectrometry (GC-MS)

**Figure 7:**
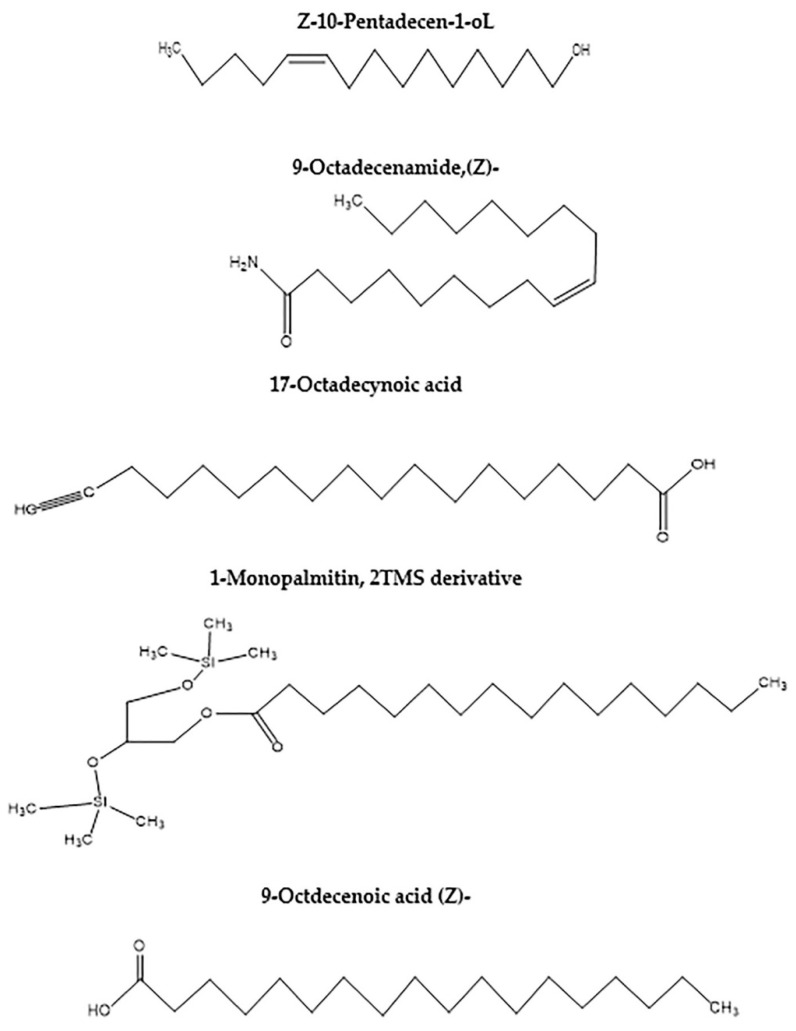
Chemical structure of the major components.

**Figure 8:**
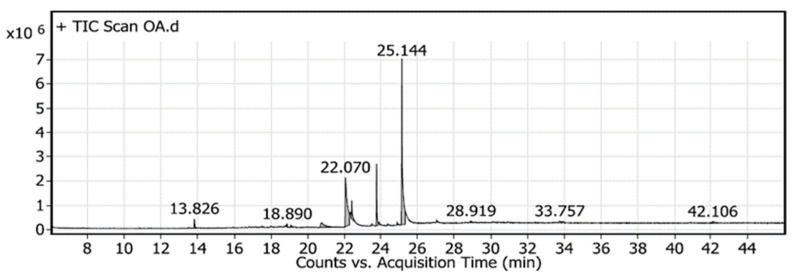
GC-MS chromatogram of fermented DPF, showing the peaks of various components.

Based on the ANN modeling process, the OA production was scaled up using the validated fermentation conditions reported above. The resulting hydrolysate of the fermented DPF recovered after SSF of DPF was explored for the various possible components using GC-MS. Twelve compounds were identified; the active principle, molecular formula (MF), concentration (peak area %), and retention time (RT) are represented in (Table 5 and Figure 7 and Figure 8). It indicates that the predominant compounds are Butylated Hydroxytoluene (1.8%), 10-Pentadecen-1-ol, (Z)-, TMS derivative (0.52%), 9-Dodecyn-1-ol, TMS derivative (0.56%), Z-10-Pentadecen-1-ol (6.06%), 9-Octadecenamide, (Z) (29.18), 17-Octadecynoic acid (4.66%), 1-Monopalmitin, 2TMS derivative (9.46%), 9-Octadecenoic acid (Z) (oleic acid (44.42%), Androstane-11,17-dione, 3-[(trimethylsilyl)oxy], 17-[O-(phenylmethyl) oxime], (3. alpha., 5. alpha.) (0.65%), Cyclobarbital (0.63%), 1,4-Bis(trimethylsilyl) benzene (0.66%) and Glycine, N-[(3α,5β)-24-oxo-3-[(trimethylsilyl)oxy] cholan-24-yl], methyl ester (1.4%). However, Z-10-Pentadecen-1-ol, 9-Octadecenamide, (Z), 17-Octadecynoic acid, 1-Monopalmitin, 2TMS derivative, and oleic acid were the major detected components in the GC-MS analysis of DPF. 

### 2.7. Ultra Performance Liquid Chromatography (UPLC) Analysis

**Figure 9:**
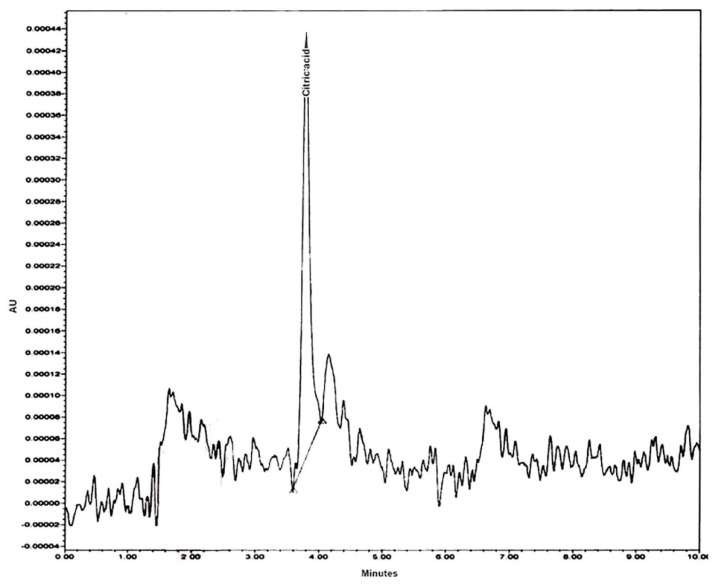
UPLC analysis of fermented DPF hydrolysate by *Trichoderma* sp. PWN6.

UPLC analysis was used to detect, identify, and quantify citric acid in the fermented DPF hydrolysate. The UPLC analysis (Figure 9) revealed an obvious accumulation titer of citric acid, being 15 mg/g DPF as the main product, which is represented by only one peak. 

### 2.8. Identification of the Fungal Strain

**Figure 10:**
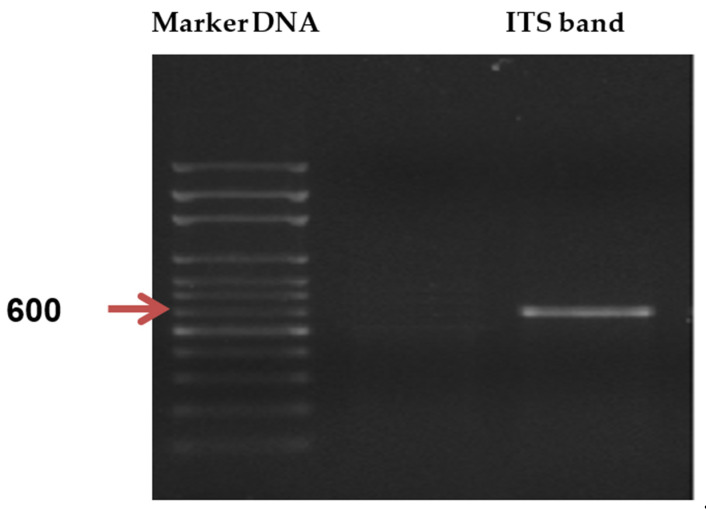
Profile of the agarose gel electrophoresis of 600 base pair band of the PCR product of the amplified ITS fragment isolated from *Trichoderma* sp. PWN6.

The selected *Trichoderma* sp. PWN6 was morphologically and molecularly identified. The morphological and microscopical investigation showed the classification of the fungus as Eukaryota; Fungi; Dikarya; Ascomycota; Pezizomycotina; Sordariomycetes; Hypocreomycetidae; Hypocreales; Hypocreaceae; *T. harzianum*. The selected fungal strain was further characterized by the molecular technique of the internal transcribed spacer (ITS) region. The PCR product of the amplified ITS fragment isolated from *Trichoderma* sp. PWN6 shows similarity to the 600 bp marker (Figure 10).

**Figure 11:**
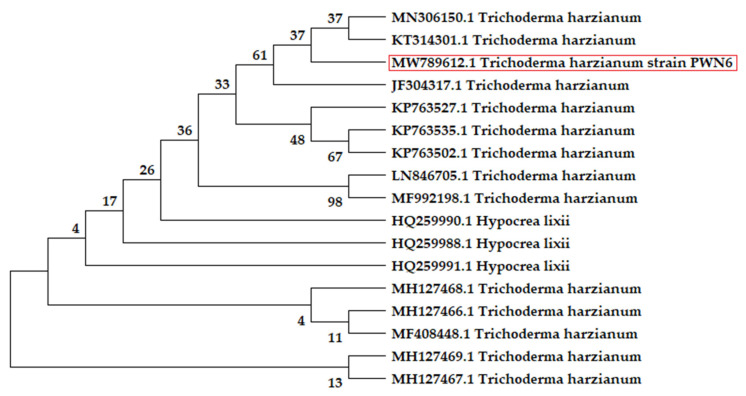
The evolutionary tree of the ITS gene’s partial sequence of *Trichoderma harzianum* strain PWN6 (located in the red rectangle) concerning the closely related sequences on the GenBank.

The BLAST analysis of the fungus candidate displayed 99% similarity with the formerly identified *Trichoderma* spp. on the Genbank. Figure 11 represents the constructed phylogenetic tree of *Trichoderma* sp. PWN6, which comes in line with the previous morphological identification as *T. harzianum*. The GenBank accession number of the present fungal strain was received as MW789612.1.

## 3. Discussion

Unfortunately, DPF is one of the natural resources that, contrary to most plant residuals, was not optimally taken advantage of yet, representing a waste of the natural wealth. The importance of microbial bioprocessing of DPF arises when the target is an important molecule, having a wide array of applications in several fields, such as organic acids (OA).

The DPF is hard to degrade tissue since it is composed mainly of cellulose and hemicellulose; therefore, the study was initiated by screening the fungal isolates for their capacity to degrade rather than grow on the DPF. The biodegradation process needs the cooperation of various enzymes. All fungal isolates were positive for the most of hydrolytic enzymes (Table 1), involving three cellulases (FPase, CMCase, and β-glucosidase) and xylanase, which can hydrolyze the cellulose and hemicellulose of plant materials, respectively. In turn, this enables microorganisms to penetrate and degrade the main components of plant tissues [11].

The main targeted component is cellulose, which is catalyzed by cellulase enzymes into single units of glucose. Several kinds of enzymes and steps are involved in the biodegradation of cellulose, in which cellulases synergistically cleave the β-1,4-glucosidic bonds in the cellulose backbone, liberating the glucose monomers [22]. This monomer is a fermentable sugar for most microorganisms [23]. By the same token, xylan is hydrolyzed by the combined action of endo-1,4-β-xylanase and β-D-xylosidases, releasing pentoses, mainly, xylose [24]. These fermentable monomers are the starting point and/or the cornerstone for the biosynthesis of various molecules, including OA, by the microorganism [22,23,24].

The SSF approach was utilized to optimize medium fermentation conditions because of its increased volumetric productivity, simplicity, reduced energy needs, ease of aeration, and simulation of the natural habitat of most fungi [25].

Analysis of the hydrolysate after SSF, obviously, revealed that the liberated glucose was negatively correlated with the amount of secreted OA. This is true since the OA formation process requires and consumes the resulted glucose for the biosynthesis of OA, reducing its amount in the hydrolysate at the expense of increasing OA production [11].

Concerning P-solubilization (Table 1), the DPF-based fermentation medium was supplemented by a complex inorganic TCP at a low level, and the fungal isolates showed a variable capacity to solubilize complex phosphate. The main reason for supporting the fermentation medium with such TCP was to induce fungi to produce OA, which mainly occurs at a low level and complex form of phosphate supply. The capacity to solubilize complex phosphates is mainly attributed to the synthesis of OA, which is produced through cellulose decomposition [9]. The solubilization process takes place by the microorganism to liberate phosphorus, which is reused for the various metabolic process, including cell growth and generation of cell energy; this process is accompanied by a significant gradual reduction in pH of the resulted filtrate and is also positively correlated with the release of OA [26]. The same pattern was found to apply in the current study. As a result, the suitable microorganism (*Trichoderma* sp. PWN6) was elected for additional investigation, i.e., optimization of the fermentation conditions toward maximization of OA biosynthesis by the selected fungus.

The next step was to determine the operational conditions that boost the bioconversion process. The recently proposed DSD (Table 2) was applied since it is a new improved class of three-level designs that can efficiently estimate main effects and two-factor interactions as well [16,18]. To the best of our knowledge, this is the first time that DSD has been utilized in the bioprocessing of plant biomass.

The Pareto chart analysis (Figure 1) and ANOVA of DSD (Table 3) show that the seven screened factors displayed a significant effect on OA biosynthesis by the selected *Trichoderma* sp. PWN6. This reflects the importance of all tested parameters in the biomass conversion process.

The overall design and the tested variables showed *p* < 0.05. According to the coefficients and *p* value, it could be concluded that a higher level of SEB and temperature, together with the lower levels of the rest of the other factors, supported the high yield of OA, which was significantly associated with changes in all seven factors. 

Other goodness-of-fit statistics were measured, of which the value of R^2^ was defined to ascertain the degree of variance in the experimental response OA values indicated by the factor(s). Adding factor(s) to the model leads to get bigger R^2^ values, even if the factor(s) was not significant. As a result, adjusted-R^2^ was employed, since it is based on the importance of the components in the model rather than their quantity. However, the higher the adjusted-R^2^, the more accurate the link between the factors and the response (OA), and hence the model fits the data effectively. Predicted-R^2^ shows how effectively the model predicts responses in new tests without over-fitting. Greater predicted-R^2^ values suggest the model’s excellent prediction efficiency. The current model explains 93.30% of the variation in OA, indicating that the model provides a good fit for the data.

To check whether the DSD model meets the assumptions of the analysis, residual analysis was performed (Figure 2). Residuals are the disparities between the experimental (observed) and the corresponding predicted value at each data point of OA. The smaller the value of the residuals, the better the fitness of the model, and hence the accuracy of the parameter selection. Depicting the normal probability plot of the residuals shows a straight line. Patterns, other than a straight line, indicate that the model does not meet the model assumptions [20]. Unusual patterns that show a non-straight line, a point that is far away from the line, or changing slope indicate non-normality, an outlier, or an unidentified variable, respectively. Furthermore, depicting the predicted vis residual showed their normal distribution along both 0-axis sides, supporting the fitness of the model. Hence, the DSD was found to be effective in optimizing the OA production, representing a new approach in the biofermentation process. Again, the alternative hypothesis, H_1_, was accepted, and data confirmed a significant effect of the tested factors on the OA biosynthesis.

As a result of the significance of all tested factors, it was suggested that artificial intelligence could be used to model the experimental DSD data. The neural network platform employs a fully connected multilayer perceptron (an algorithm for machine learning) (Figure 3). The ANN predicts response variable(s) using a flexible function of the input variable(s). The main advantages of an ANN model are the flexibility and tendency to predict the fitted data very well. Furthermore, it can model, efficiently and accurately, different response surfaces, using enough hidden nodes and layers. ANN is capable of learning any nonlinear function. Since it can learn weights that map the relationship between inputs and outputs with the aid of the activation function, that helps the network learn the complex nonlinear relationship between input and output. It is generally true that there are intermediate layers rather than a direct path from the independent variables to the response variable; therefore, ANN can be excellent predictors when it is not necessary to describe the functional form of the response surface or the relationship between the inputs and the response(s). The function applied at the nodes of the hidden layers is called the activation function, which transforms a linear combination of the various variables [20,21,27,28,29].

However, the suitable range of a factor is not fixed and changes according to the experimental situations. As an example, on the inoculum level in the current case, the inoculum of 4 × 10^7^ spore represents the half concentration of 8 × 10^7^; this range was found to be reasonably wide and accepted. The data analysis showed a high reasonably regression coefficient (−35.44) for such a factor, which was experimentally validated to be 3.5 × 10^7^ spores.

In addition, the fermentation was carried out under a restricted nutritional medium composition, which created unusual conditions for the fungal growth. Therefore, the inoculum level varied. Moreover, under the restricted nutritional composition of DPF, a high inoculum level may direct fungus to thrive for growth only and may generate a kind of fungus disturbance regarding the growth and degradation process, which may negatively affect or rerate the fungal degradation process. Lower inoculum, on the other hand, may encourage both the growth and fungal degradation to start once inoculation takes place, and that is why ammonium sulfate was incorporated and tested; i.e., to make a balance between the growth and degradation process especially at the beginning of the fermentation process.

In opposition to the prediction model, Figure 6 generates a tendency, in which the OA level decreases as the inoculum level increases. It is already known that every model had its own mechanism of mathematical calculations; however, both DSD and ANN approached the target at the same level of inoculation at the same optimum time (9 days), which confirms each other. Another tendency for the individual factor (time in the present study), when using the prediction equation, is that the factor tendency is calculated at a constant level (center point) of the other six factors. That is why some variation occurs in the tendency of incubation time. However, the final aim was achieved and validated.

Typically, the fitted model must be checked to verify that it gives an adequate approximation to the real system. Proceeding with the examination and optimization of the response surface will likely yield inadequate or misleading findings unless the model exhibits a sufficient fit. In general, residuals are used to evaluate the model’s adequacy by establishing residual values and identifying its trend. For each dataset, the residuals are shown (Figure 4) as the difference between the experimental value of OA production and its corresponding forecasted point. Checking the residuals of the ANN model prediction against the experimentally measured values of OA production shows an equal scatter of the residual data, indicating that the variance was independent of OA production, thus reinforcing the adequacy of the ANN model. Furthermore, residuals were shown to be quite minimal at all tested sites. This means that the ANN can precisely fit the real experimental data. Despite the fact that ANN modeling has made its way into various industries, there is no work on the modulation of bioconversion of DPF into OA, and this is the first work that supports this kind of modeling.

Regarding model comparison (Table 4), the ANN model was considerably better at overall classification in the prediction of OA production. The modeling capacity of a given model depends on a high R^2^ value and low RMSE, MAD, and SSE values. R^2^ evaluates the correlation between the response and anticipated values; therefore, a larger value (up to 1) indicates a significant connection between the two datasets. RMSE is commonly employed in regression analysis to authenticate experimental results since a lower value indicates that the data are concentrated around the line of best fit (prediction errors). MAD is another statistic that determines the average dispersion of data around the mean. A lower MAD value implies a reduced spread of data around the mean. Finally, SSE, another measure of goodness-of-fit, calculates the total deviation of the response values from their fitted values; a smaller number indicates that the model is more suited.

Given the facts mentioned earlier, both models demonstrated a high level of predictive ability. However, when the two models are compared, it becomes clear that DSD is lower in R^2^ and higher in the other goodness-of-fit statistics than the ANN model. As a result, the ANN model outperforms the DSD model in terms of OA production prediction. The current conclusion is conceding with a recent study [20], which reported that the ANN model was superior to RSM, recording lower RMSE, MAD, and SSE values and higher R^2^ values.

Again, another overall comparison was carried out (Figure 5), in which the linear regression analysis between the experimental values and those predicted ones was figured. The prediction points of the ANN model are substantially closer to the line of perfect prediction than those of the DSD model. So, the ANN model outperforms the DSD model in terms of generalization capability.

However, there are some merits when modeling using DSD, of which ANN modeling consumed extended computational time through many iterative calculations. Furthermore, the structured nature of the DSD can demonstrate the contributions of each factor in the regression models, thus identifying the insignificant factor(s) that can be eliminated from the model [20,28]. Anyhow, this is not applied in our case because all tested parameters had a significant effect (Figure 6). On the other side, ANN had high predictive precision due to its universal ability to approximate the system’s nonlinearity, compared with the other models, which requires only a sole step calculation for a response surface model [20,21].

Validation of the response maximization of OA production by the two models was checked and compared regarding their predictive capability. Applying the calculated theoretical values of the seven tested variables under laboratory conditions were found to yield 391.37 ± 1.38 µmol OA/g. This value is more closely related to the speculative value of ANN (387.09) than DSD (421.25). Such a result, truly, confirms the higher accuracy and predictive ability of the ANN model than the DSD one. However, and for fairness, the DSD model still has some reasonable predictive ability. 

Concerning the fermentation condition, and given the complex structure of DPF, the current study could be considered a milestone for the bioconversion of residual biomass such as DPF into citric acid in a relatively short time compared with several previous studies [8,11]. The SEB of DPF was found to facilitate the penetration of fungal hyphae into treated DPF and increase the release of nutrient contents of the biomass. The same explanation could be applied also to the particle size of DBF [30]. Both pretreatments could be used to economize and shorten the fermentation time of OA production, which was maximized after nine days only (Figure 6) in the current work, compared with several weeks in other studies [8,11].

Ammonium salts, such as AS, are simple nitrogen sources and hence required during nearly all growth stages of microorganisms; the small amount encourages the growth of the bacterium at the initial growth stage. NH_4_Cl, as an inorganic nitrogen source, was found to have a significant effect because of the simple structure; its assimilation does not need complicated biological metabolism [30]. Moreover, TCP was used as an inducer for organic acid production, this was confirmed by its significant effect during the DSD experiment. Complex phosphate solubilization by fungi is characterized by their relative ability to dissolve complex phosphates; this activity is generally related to the generation of organic acids, which are also described as end products of fungal cellulose hydrolysis [11,12].

Next to validation and the assurance of the aptness of the tested model, the hydrolysate of the fermented DPF was explored regarding the specification of major components other than CA, using GC-MS (Table 5 and Figure 7 and Figure 8) and UPLC (Figure 9). Both analyses of the hydrolysate of fermented DPF revealed the presence of various main components in addition to citric acid as one of the major OAs, confirming the hypothesis of the fermentation pathway, starting from using a complex phosphorus source in the fermentation medium to induce OA production passing by the DSD and ANN modeling, ending by the assurance of citric acid by UPLC, in which the obtained profile of citric acid indicates the presence of 1.5%. In this case, it could be said that the DPF is considered a good alternative and new for the fermentation substrate for citric acid biosynthesis, using *Trichoderma* sp. PWN6. 

The resulted citric acid is an important commercial product, and its global production is mainly consumed in the food industry (70%) of the total production, which is followed by 12% in the pharmaceutical industry and 18% for other applications [11,31].

*Trichoderma* sp. PWN6 was morphologically and molecularly identified as *T. harzianum*. The molecular identification (Figure 10 and Figure 11) came in harmony with the morphological one. For rapid and precise identification of filamentous fungi at various taxonomic levels, molecular identification techniques exhibit high accuracy, and that is why they are applied here. These methods are based on PCR amplification followed by a comparison of the gene sequence coding for 18S rRNA, during which two ITS-specific PCR primers are used. Since the fragment size of the PCR primers is consistent across many groups of fungi, nucleotide sequencing of ITS fractions is required for revealing interspecific and, in some cases, intraspecific variation [32]. Based on the well-identified sequence, the constructed phylogeny is completely annotated and shows a tight correlation with those of similar strains of *T. harzianum*. The ITS region could be used in barcode identification for different fungi, especially Basidiomycota [33]. Moreover, the ITS region is usually used and could be sufficient for fungal identification on the species level [34]. The ITS region is also considered to be among the markers with the fastest and highest probability of correct identifications for a comprehensive group of fungi [35]. 

Since the majority of the commercially produced citric acid is globally restricted to *Aspergillus* and *Penicillium* spp. [8,11,31], therefore, the new *Trichoderma harzianum* PWN6, reported in the current study, is considered a new candidate as a citric acid accumulator. This is due to the fungal capacity to ferment the complex substrate, DPF, due to its well-developed hydrolytic enzymatic system. However, a new fungal candidate that can ferment a biomass waste (DPF) on a relatively simple fermentation medium is economical enough to put such a fungal candidate on the citric acid production map.

## 4. Materials and Methods

### 4.1. Trichoderma Species

Six *Trichoderma* species (MNW1, MNW5, PWN2, PWN3, PWN4, and PWN6) were kindly provided by the Microbiology Department, Research Institute of Soils, Water, and Environment, Agricultural Research Center, Giza, Egypt. 

### 4.2. SEB

Date palm fronds (*Phoenix dactylifera* of type, Zaghoul) were collected during September 2019 from the Agricultural Research Center, Cairo, Egypt (30°01′13.8″ N and 31°12′15.8” E). The DPF was washed several times to remove dirt, the last was done by deionized water and dried in a shaded area; then, it was collected, cut, and milled before chemical analysis. The main chemical components were carbohydrates (19.95%), fats (2.70%), protein (10.00%), hemicellulose (7.52%), cellulose (34.55%), lignin (9.28%), and ash (6.00%). The total organic matter was 84.00%. The level of macroelements N, P, and K were 1.60, 0.12, and 3.48 mg/kg, respectively, and the microelements were Fe, Cu, and Mn, being 240.20, 1.03, and 76.73 mg/kg, respectively. The ground particles were graded, by gradient sieves, to various sizes, i.e., 2, 4, and 6 mm long, which were confirmed again by vernier caliper. A leaflet of DPF was applied to serve as solid support and substrate for OA production.

The residues of DPF were pretreated with a steam explosion procedure using an autoclave. For this process, 300 g of the biomass was pretreated by autoclave under pressure at 15 pounds per square inch (psi), the temperature was maintained at 121 °C for 15 min. Then, the under pressured-steam was allowed to suddenly discharge from the autoclave; the resultant was SEB of DPF.

### 4.3. Fermentation Medium

The ability of fungal isolates to ferment palm fronds was evaluated using SSF. The fermentation medium contained one gram of DPF in flasks (100 mL). The fermentation medium was supplemented with 15 mg from each TCP and (NH_4_)_2_SO_4_. Sterilization was carried out at 121 °C for 20 min.

### 4.4. Fermentation Procedure

Unless otherwise specified, before the fermentation trial, the fungal inoculum was prepared freshly from 5 days-aged culture through scraping against distilled tap water to obtain an inoculum of 10^7^ spore ml^−1^ using a hemocytometer. A known concentration of the spore suspension of each fungus was inoculated into the previous medium, which was followed by incubation at 28 °C for 7 days. The moisture content was adjusted to about 65%. After the fermentation period, the fermented matter was eluted using 10 mL of distilled water. The hydrolysate was used for biochemical analysis.

### 4.5. Modeling of OA Biosynthesis

#### 4.5.1. Constructing the DSD

The purpose was to investigate the relative importance and significance of each tested variable of fermentation conditions. To accommodate the DSD structure, seven independent variables (Table 1) were tested at two corner points (low (−1), and high (+1) levels) and one center (0) level for each factor. The range of the tested parameters was selected based on a preliminary experiment to cover the proposed range. The center points indicate experimental runs in which all factor values were set midway between low and high settings. One of the selected factors was two-level categorical i.e., SEB-DPF (L1) and no-SEB (L2), having only two levels. The other six factors were all of three-level, numeric and continuous. They were DPF size (2, 4, 6 mm long), incubation time (5, 7, and 9 day), incubation temperature (25, 30, and 35 °C), inoculation size (4, 6, and 8 × 10^7^ spore/g DPF), TCP (10, 15, and 20 mg/g DPF), and AS (10, 15, and 20 mg/g DPF). Accordingly, a base matrix of 18 experimental runs was generated with three replicates each, yielding a total of 54 runs.

The SSF technique was applied for screening and optimizing the fermentation factors affecting the biosynthesis of OA. The SSF was implemented by fermenting one gram of ground DPF in a 100-mL Erlenmeyer flask, following the various combinations of fermentation design reported in the DSD matrix. The moisture of the fermented matter was kept at about 65% by moistening with sterilized tap water when needed. After the fermentation period, the fermented DPF was agitated for 30 min at 200 rev./min on a rotary shaker at room temperature with 10 volumes of distilled water containing 0.25% of brej 35 as a surfactant. The fermented biomass was then separated by filtration followed by centrifugation at 5000 rev./min for 15 min and the filtrate was assayed for OA.

#### 4.5.2. ANN for Modeling OA Biosynthesis

The previous DSD matrix, along with the corresponding data, were used to feed ANN. A fully connected neural networks platform was constructed with one hidden layer; all nodes within the layer have the same hyperbolic tangent sigmoid activation function (NTanH). Experimental data obtained from the DSD matrix were employed to train the artificial neural network and create the prediction model using a fully connected multilayer perceptron algorithm. The data were portioned, randomly, into three datasets, the first for training (using 36 runs to minimize prediction error and establish neural weights, the second for validation (using 18 runs to stop ANN training and selection of the best model, with a holdback propagation of 0.3333), and the third is an external dataset used for testing ANN robustness: i.e., the final assessment of prediction capabilities. The latter dataset was not used in model selection, and it was excluded during model development and used only for final assessment. The neural network had three layers. The ANN topology was designated as 7-h-1. The input layer was composed of seven neurons (SEB, DPF size (mm), incubation time (day), incubation temperature (°C), inoculation (spore/g), TCP (mg/g), and AS (mg/g)), and the output layer has one neuron (OA production, µmol/g)). Between the two layers, another hidden layer was constructed and tested using a number of neurons ranging from 3 to 10. The penalty method at a learning rate of 0.1 was used for fitting the model with 100 tours, using the trial-and-error procedure to train the ANN. Once the minimum values of RMSE, MAD, and SSE were reached, accompanied by the highest value of the R^2^, the predicted outputs were extremely near to the real OA production’s response target value.

### 4.6. Biochemical Analysis

#### 4.6.1. Colorimetrical Determinations

Assay of cellulases in the post-culture filtrate was carried out following the procedures of [11,36], with slight modification, in which the activities of FPase, CMCase, β-glucosidase, and xylanase on 1% of microcrystalline cellulose, carboxymethyl cellulose, cellobiose, and oat-spelled xylan (Sigma-Aldrich) were assayed, respectively. All substrates were individually dissolved in citrate buffer (0.05 M, pH 4.8); the reaction mixture (1 mL of the filtrate and 1 mL appropriate substrate buffer solution) was incubated at 50 °C for 60, 30, 15, and 30 min, respectively. The released reducing sugars by the enzymatic action was determined by the dinitro salicylic acid method [37]. Enzyme unit (U) is defined as the amount of enzyme required to release one µmol min-1 of glucose (FPase or CMCase or β-glucosidase) or xylose (xylanase) under the test conditions.

Glucose determination was carried out in the post-culture filtrate. The released glucose monomers due to the fermentation were determined using a glucose oxidase kit (Spainreact Co., Spain). The free soluble phosphorus released as a result of the biodegradation was measured in the post-culture filtrate [38].

The colorimetrical determination of the total OA in the filtrate was performed based on the method described by [39]. The pH of the post-culture filtrate was measured using the glass electrode pH meter (CP-501, Elmetron).

#### 4.6.2. GC-MS Analysis

The samples were extracted and resuspended in 50 µL of BSTFA incubated in a Dry Block Heater at 70 °C for 30 min. GC-MS analysis of the hydrolysate of DPF was performed using the GC-MS system (Agilent Technologies) that was equipped with a gas chromatograph (7890B) and mass spectrometer detector (5977A). The GC was equipped with an HP-5MS column (30 m × 0.25 mm internal diameter and 0.25 μm film thickness). Analysis was carried out using hydrogen as the carrier gas at a flow rate of 1.0 mL/min at a split-less, injection volume of 2 µL, and the temperature was programmed to 50 °C for 1 min, with a rising rate at 10 °C/min, up to 300 °C, and held for 20 min. The injector and detector were held at 250 °C. Mass spectra were obtained by electron ionization at 70 eV, using a spectral range of 30–700 *m*/*z* and solvent delay of 9 min. The mass temperature was 230 °C, and the Quad temperature was 150 °C. Different elements were identified by matching the spectrum fragmentation pattern to those contained in Wiley and NIST Mass Spectral Library data.

#### 4.6.3. UPLC-PDA Analysis and Quantification of CA

The identification and quantification of organic acid are commonly performed by Liquid Chromatography. The organic acids chromatographic profile of the fermented DPF extract was performed using a Waters UPLC Acquity H Class (Waters, Milford, MA, USA) equipped with a quaternary pump (UPQSM), autosampler injector, and a photodiode array detector (PDA). Empower 3 software (Waters, 2010, Milford, MA, USA) was used for data acquisition and processing. Chromatographic separation of the citric acid was carried out using a Waters Acquity UPLC Spherisorb at room temperature with a linear flow rate of 0.8 mL/min and on water Atlantis T3 C18 column (4.6 × 250 mm × 5 µm) with 0.01 mol/L sulfuric acid in waters as mobile phase (isocratic elution); PDA was set at a wavelength of 220 nm.

### 4.7. Identification of Fungal Strain

The selected *Trichoderma* sp. PWN6 was morphologically and molecularly identified. The complete morphological characterization was done on the selected fungus by observing the growth character of the fungus on agar plates. The measurements and examinations of the morphological structures and vegetative mycelia were investigated under a light microscope by mounting portions of fungal growth in a lactophenol cotton blue stain on clean slides [40,41,42].

The molecular identification protocol was performed. The polymerase chain reaction (PCR) amplification was performed in a total volume of 50 uL, containing 1x reaction buffer, 1.5 mM MgCl2, 1U Taq DNA polymerase (Promega), 2.5mM dNTPs, 30 picomoles of each primer (ITS-1 F (5′-TCCGTAGGTGAACCTGCGG-3′) and ITS4 R (5′-TCCTCCGCTTATTGATATGC-3′)), and 30 ng genomic DNA. Thermo-cycling PCR program for PCR amplification was performed in a Perkin-Elmer/GeneAmp^®^ PCR System 9700 (PE Applied Biosystems) programmed to fulfill 40 cycles after an initial denaturation cycle for 5 min at 94 °C. Each cycle consisted of a denaturation step at 94 °C for 30 s, an annealing step at 45 °C for 30 s, and an elongation step at 72 °C for 1 min. The primer extension segment was extended to 7 min at 72 °C in the final cycle. The amplified product of the PCR was resolved by electrophoresis in a 1.5% agarose gel containing ethidium bromide (0.5 µg/mL) in 1x TBE buffer at 95 volts. A 100 bp DNA ladder was used as a molecular size standard. PCR products were visualized on UV light and photographed using a Gel Documentation System (BIO-RAD 2000). The amplified product was purified using EZ-10 spin column PCR products; the purification PCR reaction mixture was transferred to a 1.5 mL microfuge tube, and three volumes were added of binding buffer 1. After that, the mixture solution was transferred to the EZ-10 column and left to stand at room temperature for 2 min. After that centrifuge, 750 uL of wash solution was added to the column and centrifuged at 10,000 rpm for two minutes, after which repeated washing at 10,000 rpm was conducted for an additional minute to remove any residual wash solution. The column was transferred into a clean 1.5 mL microfuge tube, and 50 uL of elution buffer was added, incubated at room temperature for 2 min, and purified DNA was stored at −20 °C. 

The ITS sequencing analysis of the PCR product was carried through in an automatic sequencer ABI PRISM 3730XL Analyzer using Big Dye TM Terminator Cycle Sequencing Kits, following the protocols supplied by the manufacturer. Single-pass sequencing was performed on each template using Rbcl Forward primer. The fluorescent-labeled fragments were purified from the unincorporated terminators with an ethanol precipitation protocol. The samples were resuspended in distilled water and subjected to electrophoresis in an ABI 3730xl sequencer (Microgen Company). The ITS sequence (851 bp) was computationally analyzed using the BLASTn program (http://www.ncbi.nlm.nih.gov/BLAST). Sequences were aligned using Align Sequences Nucleotide BLAST. The obtain sequence has been deposited in GenBank to obtain the closely related fungi sequences; then, the accession number of the fungal strain was received. The evolutionary relationship was deduced using the Neighbor-Joining method. The bootstrap consensus tree was inferred from 2000 replicates. The evolutionary distances were computed using the Jukes–Cantor method and are in the units of the number of base substitutions per site. This analysis involved 17 nucleotide sequences. All ambiguous positions were removed for each sequence pair (pairwise deletion option). MEGA 10 software was used to conduct the evolutionary analyses.

### 4.8. Trial Design and Statistical Examination

The results of the measured biodegradability of DPF by *Trichoderma* spp. are expressed as mean ± SD of three biological replicates, with the aid of CoStat 6.4 software (IBM Corporation, Armonk, New York, USA). The design and statistical analysis of DSD were accomplished using the Minitab statistical analysis software package (version 19.2, Minitab Inc., State College, PA, USA) software. Experiments of DSD were repeated thrice. The ANN topology and model comparison were set up using the JMP Pro statistical analysis software package (JMP Pro.^®^, Version 14.3. SAS Institute Inc., Cary, NC, USA, 1989–2019). Training of ANN was performed using 36 erratically chosen runs by the software, whereas the other 18 runs were used to check the validity of the trained ANN model.

## 5. Conclusions

Summing up, several merits could be extracted from the current study. Firstly, the majority of the previous studies dealt with the remediation of plant biomass through two sequenced steps: initiating by the saccharification of plant biomass into single monomers, then fermenting the monomers into the target molecule. This study, on the other side, suggests a single-step bioconversion of plant biomass into citric acid in a relatively short time (9 days) in relation to the complex structure of the DPF. Secondly, this is the first study that uses both DSD together with ANN for modeling the bioconversion process of residual biomass into a valuable biomolecule i.e., citric acid, using a new *Trichoderma* candidate. Therefore, the current study could be considered a milestone for applying artificial intelligence on biomass remediation in the upcoming studies of similar areas, representing a base for applying artificial intelligence in future research discoveries to highlight such virgin areas.

## Figures and Tables

**Table 1:** Screening the cellulolytic activity and the bioconversion ability of DPF into organic acids by various *Trichoderma* spp. (mean ± SD).

Isolate	Glucose (µmol/g)	U (µmol/g/min)				OA (µmol/g)	Filtrate pH	Soluble P (mg/g)
		FPase	CMCase	β-Glucosidase	Xylanase			
*Trichoderma* sp. MNW1	2463.0 ± 25.3	87.26 ± 7.7	0.16 ± 0.02	367.80 ± 17.6	38.44 ± 7,8	170.20 ± 13.4	5.22 ± 0.3	6.41 ± 0.8
*Trichoderma* sp. MNW5	2284.6 ± 15.7	88.33 ± 6.8	0.16 ± 0.01	411.61 ± 17.2	39.37 ± 8.3	170.78 ± 8.8	5.49 ± 0.4	5.67 + 0.7
*Trichoderma* sp. PWN2	2752.7 ± 33.8	40.51 ± 12.1	0.00	130.76 ± 28.4	29.31 ± 3.8	0.00	5.94 ± 0.4	2.01 ± 0.3
*Trichoderma* sp. PWN3	2117.5 ± 39.1	85.94 ± 4.9	0.05 ± 0.01	290.50 ± 13.5	50.07 ± 6.6	28.40 ± 3.3	5.43 ± 0.2	4.36 ± 0.8
*Trichoderma* sp. PWN4	2485.3 ± 27.4	32.25 ± 5.2	0.00	231.06 ± 9.3	50.25 ± 5.2	3.32 ± o.9	5.78 ± 0.1	3.28 ± 0.5
*Trichoderma* sp. PWN6	1783.1 ± 13.5	93.03 ± 5.6	0.18 ± 0.03	425.72 ± 19.7	51.53 ± 4.5	195.41 ± 9.7	5.13 ± 0.2	7.63 ± 0.9
Simple correlation coefficient								
FPase	−0.715 ^NS^							
CMCase	−0.615 ^NS^	0.875 **						
β-glucosidase	−0.747 ^NS^	0.865 **	0.946 **					
Xylanase	−0.752 ^NS^	0.240 ^NS^	0.133 ^NS^	0.411 ^NS^				
OA (µmol/g)	−0.566 ^NS^	0.807 ^NS^	0.992 **	0.922 **	0.097 ^NS^			
Filtrate pH	0.777 ^NS^	−0.893 **	−0.867 **	−0.887 **	−0.453 ^NS^	−0.834 **		
Soluble P (mg/g)	−0.769 ^NS^	0.852 **	0.945 **	0.955 **	0.406 ^NS^	0.935 **	−0.959 **	
	Glucose (µmol/g)	FPase	CMCase	β-glucosidase	Xylanase	OA (µmol/g)	Filtrate pH	Soluble P (mg/g)

Date palm fronds (4 mm size) did not receive any pretreatment to simulate the natural growth conditions of the fungi. NS, non-significant; **, significant at *p* ≤ 0.05.

**Table 2:** Data of OA production generated based on the design matrix of DSD of the independent variables and the equivalent predicted values forecasted by DSD and ANN models.

Run	SEB	DPF Size (mm)	Time (Day)	Temperature (°C)	Inoculation (×10^7^ spore/g)	TCP (mg/g)	AS (mg/g)	OA (µmol/g)				
								Actual	DSD		ANN	
									Fitted	Residual	Fitted	Residual
1	L1 (−1)	6 (+1)	9 (+1)	35 (+1)	8 (+1)	20 (+1)	20 (+1)	222.33	209.77	12.56	213.22	9.11
2 *	L2 (+1)	2 (−1)	5 (−1)	25 (−1)	4 (−1)	10 (−1)	10 (−1)	330.21	336.87	−6.66	344.69	−14.48
3	L2 (+1)	4 (0)	9 (+1)	25 (−1)	8 (+1)	10 (−1)	10 (−1)	240.65	235.52	5.13	236.61	4.04
4	L1 (−1)	4 (0)	5 (−1)	35 (+1)	4 (−1)	20 (+1)	20 (+1)	283.76	311.12	−27.36	315.60	−31.84
5 *	L2 (+1)	2 (−1)	7 (0)	25 (−1)	8 (+1)	20 (+1)	20 (+1)	170.28	166.87	3.41	158.65	11.63
6 *	L1 (−1)	6 (+1)	7 (0)	35 (+1)	4 (−1)	10 (−1)	10 (−1)	372.49	379.77	−7.28	378.04	−5.55
7 *	L2 (+1)	6 (+1)	9 (+1)	30 (0)	4 (−1)	20 (+1)	10 (−1)	301.11	299.76	1.35	318.41	−17.30
8	L1 (−1)	2 (−1)	5 (−1)	30 (0)	8 (+1)	10 (−1)	20 (+1)	240.41	246.88	−6.47	250.11	−9.70
9	L2 (+1)	2 (−1)	5 (−1)	35 (+1)	6 (0)	20 (+1)	10 (−1)	341.26	367.89	−26.63	355.06	−13.80
10 *	L1 (−1)	6 (+1)	9 (+1)	25 (−1)	6 (0)	10 (−1)	20 (+1)	152.84	178.74	−25.90	172.37	−19.53
11	L2 (+1)	6 (+1)	5 (−1)	25 (−1)	4 (−1)	15 (0)	20 (+1)	200.61	225.77	−25.16	216.49	−15.88
12	L1 (−1)	2 (−1)	9 (+1)	35 (+1)	8 (+1)	15 (0)	10 (−1)	300.00	320.87	−20.87	315.87	−15.87
13	L2 (+1)	6 (+1)	5 (−1)	35 (+1)	8 (+1)	10 (−1)	15 (0)	311.64	309.50	2.14	314.25	−2.61
14	L1 (−1)	2 (−1)	9 (+1)	25 (−1)	4 (−1)	20 (+1)	15 (0)	223.27	237.14	−13.87	239.23	−15.96
15	L2 (+1)	2 (−1)	9 (+1)	35 (+1)	4 (−1)	10 (−1)	20 (+1)	385.64	381.36	4.28	372.78	12.86
16	L1 (−1)	6 (+1)	5 (−1)	25 (−1)	8 (+1)	20 (+1)	10 (−1)	149.46	165.27	−15.81	160.43	−10.97
17	L1 (−1)	4 (0)	7 (0)	30 (0)	6 (0)	15 (0)	15 (0)	260.29	262.83	−2.53	262.56	−2.27
18	L2 (+1)	4 (0)	7 (0)	30 (0)	6 (0)	15 (0)	15 (0)	297.28	283.81	13.47	292.09	5.19
19 *	L1 (−1)	6 (+1)	9 (+1)	35 (+1)	8 (+1)	20 (+1)	20 (+1)	228.27	209.77	18.50	213.22	15.05
20	L2 (+1)	2 (−1)	5 (−1)	25 (−1)	4 (−1)	10 (−1)	10 (−1)	353.26	336.87	16.39	344.69	8.57
21	L2 (+1)	4 (0)	9 (+1)	25 (−1)	8 (+1)	10 (−1)	10 (−1)	220.81	235.52	−14.71	236.61	−15.80
22 *	L1 (−1)	4 (0)	5 (−1)	35 (+1)	4 (−1)	20 (+1)	20 (+1)	351.24	311.12	40.12	315.60	35.64
23	L2 (+1)	2 (−1)	7 (0)	25 (−1)	8 (+1)	20 (+1)	20 (+1)	141.85	166.87	−25.02	158.65	−16.80
24	L1 (−1)	6 (+1)	7 (0)	35 (+1)	4 (−1)	10 (−1)	10 (−1)	351.46	379.77	−28.31	378.04	−26.58
25	L2 (+1)	6 (+1)	9 (+1)	30 (0)	4 (−1)	20 (+1)	10 (−1)	314.47	299.76	14.71	318.41	−3.94
26	L1 (−1)	2 (−1)	5 (−1)	30 (0)	8 (+1)	10 (−1)	20 (+1)	266.61	246.88	19.73	250.11	16.50
27	L2 (+1)	2 (−1)	5 (−1)	35 (+1)	6 (0)	20 (+1)	10 (−1)	371.80	367.89	3.91	355.06	16.74
28	L1 (−1)	6 (+1)	9 (+1)	25 (−1)	6 (0)	10 (−1)	20 (+1)	190.84	178.74	12.10	172.37	18.47
29 *	L2 (+1)	6 (+1)	5 (−1)	25 (−1)	4 (−1)	15 (0)	20 (+1)	220.29	225.77	−5.48	216.49	3.80
30	L1 (−1)	2 (−1)	9 (+1)	35 (+1)	8 (+1)	15 (0)	10 (−1)	321.53	320.87	0.66	315.87	5.66
31 *	L2 (+1)	6 (+1)	5 (−1)	35 (+1)	8 (+1)	10 (−1)	15 (0)	320.37	309.50	10.87	314.25	6.12
32 *	L1 (−1)	2 (−1)	9 (+1)	25 (−1)	4 (−1)	20 (+1)	15 (0)	244.42	237.14	7.28	239.23	5.19
33 *	L2 (+1)	2 (−1)	9 (+1)	35 (+1)	4 (−1)	10 (−1)	20 (+1)	361.88	381.36	−19.48	372.78	−10.90
34	L1 (−1)	6 (+1)	5 (−1)	25 (−1)	8 (+1)	20 (+1)	10 (−1)	178.14	165.27	12.87	160.43	17.71
35	L1 (−1)	4 (0)	7 (0)	30 (0)	6 (0)	15 (0)	15 (0)	264.21	262.83	1.38	262.56	1.65
36	L2 (+1)	4 (0)	7 (0)	30 (0)	6 (0)	15 (0)	15 (0)	298.65	283.81	14.84	292.09	6.56
37	L1 (−1)	6 (+1)	9 (+1)	35 (+1)	8 (+1)	20 (+1)	20 (+1)	200.64	209.77	−9.13	213.22	−12.58
38	L2 (+1)	2 (−1)	5 (−1)	25 (−1)	4 (−1)	10 (−1)	10 (−1)	361.47	336.87	24.60	344.69	16.78
39 *	L2 (+1)	4 (0)	9 (+1)	25 (−1)	8 (+1)	10 (−1)	10 (−1)	229.98	235.52	−5.54	236.61	−6.63
40 *	L1 (−1)	4 (0)	5 (−1)	35 (+1)	4 (−1)	20 (+1)	20 (+1)	302.46	311.12	−8.66	315.60	−13.14
41 *	L2 (+1)	2 (−1)	7 (0)	25 (−1)	8 (+1)	20 (+1)	20 (+1)	159.23	166.87	−7.64	158.65	0.58
42 *	L1 (−1)	6 (+1)	7 (0)	35 (+1)	4 (−1)	10 (−1)	10 (−1)	380.54	379.77	0.77	378.04	2.50
43	L2 (+1)	6 (+1)	9 (+1)	30 (0)	4 (−1)	20 (+1)	10 (−1)	315.82	299.76	16.06	318.41	−2.59
44 *	L1 (−1)	2 (−1)	5 (−1)	30 (0)	8 (+1)	10 (−1)	20 (+1)	260.18	246.88	13.30	250.11	10.07
45 *	L2 (+1)	2 (−1)	5 (−1)	35 (+1)	6 (0)	20 (+1)	10 (−1)	350.66	367.89	−17.23	355.06	−4.40
46	L1 (−1)	6 (+1)	9 (+1)	25 (−1)	6 (0)	10 (−1)	20 (+1)	171.83	178.74	−6.91	172.37	−0.54
47	L2 (+1)	6 (+1)	5 (−1)	25 (−1)	4 (−1)	15 (0)	20 (+1)	219.77	225.77	−6.00	216.49	3.28
48	L1 (−1)	2 (−1)	9 (+1)	35 (+1)	8 (+1)	15 (0)	10 (−1)	323.66	320.87	2.79	315.87	7.79
49 *	L2 (+1)	6 (+1)	5 (−1)	35 (+1)	8 (+1)	10 (−1)	15 (0)	310.17	309.50	0.67	314.25	−4.08
50	L1 (−1)	2 (−1)	9 (+1)	25 (−1)	4 (−1)	20 (+1)	15 (0)	251.86	237.14	14.72	239.23	12.63
51	L2 (+1)	2 (−1)	9 (+1)	35 (+1)	4 (−1)	10 (−1)	20 (+1)	382.46	381.36	1.10	372.78	9.68
52	L1 (−1)	6 (+1)	5 (−1)	25 (−1)	8 (+1)	20 (+1)	10 (−1)	160.95	165.27	−4.32	160.43	0.52
53	L1 (−1)	4 (0)	7 (0)	30 (0)	6 (0)	15 (0)	15 (0)	283.44	262.83	20.61	262.56	20.88
54	L2 (+1)	4 (0)	7 (0)	30 (0)	6 (0)	15 (0)	15 (0)	310.46	283.81	26.65	292.09	18.37

Date palm fronds (DPF) were tested at two levels; SEB (steam-exploded biomass)-DPF (L1) and no-SEB (L2), the number between parenthesis is the corresponding coded value of the tested parameters. TCP, tricalcium phosphate; AS, (NH_4_)_2_SO_4_. The program software, randomly, chooses 36 trials for training the ANN, while the remaining 18 trials (*) were utilized for validation.

**Table 3:** Regression coefficient (coded units) and analysis of variance of the total organic acids produced by *Trichoderma* sp. PWN6. based on the experimental data of the DSD matrix.

Source	Regression Coefficient	Freedom Degree	Contribution, %	Sum of Square	Mean Square	F-Value	*p*-Value
Model	273.32	7	95.13	250772	35,825	128.38	<0.001 *
Linear		7	95.13	250,772	35,825	128.38	<0.001 *
SEB	10.49	1	5.50	5379	5379	19.28	<0.001 *
DPF size	−19.17	1	5.44	15,161	15,161	54.33	<0.001 *
Time	−5.65	1	0.42	1319	1319	4.73	0.035 *
Temperature	53.93	1	47.93	120,063	120,063	430.24	<0.001 *
Inoculation	−35.44	1	18.81	51,835	51,835	185.75	<0.001 *
TCP	−20.7	1	6.42	17,691	17,691	63.39	<0.001 *
AS	−26.03	1	10.61	27,972	27,972	100.24	<0.001 *
Error		46	4.87	12,837	279		
Lack-of-fit		10	1.68	4429	443	1.90	0.078 ^NS^
Pure error		36	3.19	8408	234		
Total		53	100	766,638			
The goodness-of-fit statistics of the model							
Standard deviation				16.7051			
Coefficient of determination (R^2^)				0.951			
Adjusted-R^2^				0.944			
Predicted-R^2^				0.933			
Predicted residual error sum of squares				17,654.7			

SEB, steam-exploded biomass; TCP, tricalcium phosphate, Ca_3_(PO_4_)_2_; AS, ammonium sulfate (NH_4_)_2_SO_4_; NS, non-significant; *, significant at *p* ≤ 0.05.

**Table 4:** Statistics of the model comparison generated by DSD and ANN.

Training Statistics				
**Model**	**R^2^**	**RMSE**	**MAD**	**Number of Used Runs**
DSD	0.947	15.71	13.16	36
ANN	0.960	13.58	11.41	36
**Validation Statistics**				
DSD	0.958	14.82	11.12	18
ANN	0.967	13.15	10.37	18
**Overall Model Comparison**				
**Statistics**		**DSD**	**ANN**	**Number of Used Runs**
R^2^		0.951	0.963	54
RMSE		15.42	13.44	54
MAD		12.48	11.06	54
SSE		12836.7	9749.5	54

Root mean squared error (RMSE), mean absolute deviation (MAD), the sum of squares due to error (SSE).

**Table 5:** GC-MS differentiation of the compounds detected in the hydrolysate of the fermented DPF by *Trichoderma* sp. PWN6.

Peak	RT	Name	Formula	Area	Area Sum %
1	13.826	Butylated Hydroxytoluene	C_15_H_24_O	920,577.74	1.8
2	18.89	10-Pentadecen-1-ol, (Z), TMS derivative	C_18_H_38_OSi	265,128.93	0.52
3	19.093	9-Dodecyn-1-ol, TMS derivative	C_15_H_30_OSi	286,143.94	0.56
4	20.759	Z-10-Pentadecen-1-ol	C_15_H_30_O	3,106,296.7	6.06
5	22.07	9-Octadecenamide, (Z)	C_18_H_35_NO	14,953,644	29.18
6	22.409	17-Octadecynoic acid	C_18_H_32_O_2_	2,387,210	4.66
7	23.765	1-Monopalmitin, 2TMS derivative	C_25_H_54_O_4_Si_2_	4,850,255.3	9.46
8	25.144	9-Octadecenoic acid (Z)	C_18_H_34_O_2_	22,763,917	44.42
9	28.919	Androstane-11,17-dione, 3-[(trimethylsilyl)oxy], 17-[O-(phenylmethyl)oxime], (3. alpha.,5. alpha.)	C_29_H_43_NO_3_Si	334,647.18	0.65
10	33.757	Cyclobarbital	C_12_H_16_N_2_O_3_	321,345.1	0.63
11	33.908	1,4-Bis(trimethylsilyl)benzene	C_12_H_22_Si_2_	340,266.45	0.66
12	42.106	Glycine, N-[(3α,5β)-24-oxo-3-[(trimethylsilyl)oxy]cholan-24-yl], methyl ester	C_30_H_53_NO_4_Si	719,139.75	1.4

## Sample Availability

Samples of the compounds are not available from the authors.
